# Polyhydroxyalkanoate (PHA) Biopolymer Synthesis by Marine Bacteria of the Malaysian Coral Triangle Region and Mining for PHA Synthase Genes

**DOI:** 10.3390/microorganisms10102057

**Published:** 2022-10-18

**Authors:** Athraa Alsaadi, Sree Selva Kumar Ganesen, Tan Suet May Amelia, Radwa Moanis, Eveline Peeters, Sevakumaran Vigneswari, Kesaven Bhubalan

**Affiliations:** 1 Department of Biology, Faculty of Math, Physics and Natural Sciences, Universita degli Studi di Firenze (UniFi), 50121 Firenze, Italy; 2 Research Group of Microbiology, Department of Bioengineering Sciences, Vrije Universiteit Brussel (VUB), 1050 Brussels, Belgium; 3 Faculty of Science and Marine Environment, Universiti Malaysia Terengganu, Kuala Nerus 21030, Malaysia; 4 Faculty of Science, Damanhour University, Damanhour 5842001, Egypt; 5 Institute of Marine Biotechnology, Universiti Malaysia Terengganu, Kuala Nerus 21030, Malaysia

**Keywords:** bioplastic, PHA synthase, phaC, sediment, marine sponge, diversity

## Abstract

Polyhydroxyalkanoate (PHA), a biodegradable and plastic-like biopolymer, has been receiving research and industrial attention due to severe plastic pollution, resource depletion, and global waste issues. This has spurred the isolation and characterisation of novel PHA-producing strains through cultivation and non-cultivation approaches, with a particular interest in genes encoding PHA synthesis pathways. Since sea sponges and sediment are marine benthic habitats known to be rich in microbial diversity, sponge tissues (*Xestospongia muta* and *Aaptos aaptos*) and sediment samples were collected in this study from Redang and Bidong islands located in the Malaysian Coral Triangle region. PHA synthase (*phaC*) genes were identified from sediment-associated bacterial strains using a cultivation approach and from sponge-associated bacterial metagenomes using a non-cultivation approach. In addition, phylogenetic diversity profiling was performed for the sponge-associated bacterial community using 16S ribosomal ribonucleic acid (16S rRNA) amplicon sequencing to screen for the potential presence of PHA-producer taxa. A total of three *phaC* genes from the bacterial metagenome of *Aaptos* and three *phaC* genes from sediment isolates (*Sphingobacterium mizutaii* UMTKB-6, *Alcaligenes faecalis* UMTKB-7, *Acinetobacter calcoaceticus* UMTKB-8) were identified. Produced PHA polymers were shown to be composed of 5C to *n*C monomers, with previously unreported PHA-producing ability of the *S. mizutaii* strain, as well as a 3-hydroxyvalerate-synthesising ability without precursor addition by the *A. calcoaceticus* strain.

## 1. Introduction

Petroleum-derived plastics are globally and rapidly utilised materials for daily usage. With the rapid growth of the human population, the dependency on the material, and the coronavirus disease 2019 (COVID-19) pandemic, plastic waste generation escalated drastically despite national and international efforts in policy implementation [1]. Plastic is produced from fossil fuel resources, degrades very slowly, emits toxic gases when decomposing, and carries pollutants, especially when in the form of micro-sized fragments (i.e., microplastics) [2]. Due to the hazards of plastics and international agendas such as the UN Sustainable Development Goals (SDGs), plastic pollution and awareness have gradually shifted the focus of scientific and industrial communities towards a sustainable economy and alternative approaches, such as discovering environmentally friendly substitutes that are plastic-like materials [3].

A class of biodegradable polymers that closely mimics the properties of petroleum-derived plastics are polyhydroxyalkanoates (PHAs), which are microbially produced and biodegradable polyesters consisting of hydroxycarboxylic acids [4]. PHAs are naturally produced by a broad diversity of prokaryotic microorganisms, including bacteria. PHA synthesis is induced as a survival response in stressful environmental conditions, in the presence of excess carbon and limited other nutrients, such as nitrogen and phosphorus [5]. The polymer accumulates as insoluble granules within the cytoplasm as long-term carbon and energy storage for survival. The polymer intracellularly accumulates in the form of insoluble granules. These PHA granules could be considered long-term carbon and energy storage. When left in the environment, PHA is microbially decomposed into carbon dioxide and water [6].

PHA polymers are categorised based on their carbon atom number and their monomer composition, which influence their physicochemical properties (e.g., elongation-to-break and crystallisation characteristics). PHAs with monomers of three to five carbon atoms are classified as short-chain-length (SCL) PHAs and monomers of six to fourteen carbon atoms as medium-chain-length (MCL) PHAs. PHAs with a combination of SCL and MCL monomers are known as SCL-MCL PHA heteropolymers [3,5]. SCL PHAs are generally brittle, while the elastomeric MCL PHAs or copolymers with a higher fraction of MCL monomers have a lower crystallisation degree (20–40%) and a higher elongation-to-break parameter (300–450%); hence, they are more chemically flexible and modifiable to suit diverse applications [3,7].

The sustainable and advantageous properties of PHA have attracted research on uncovering novel PHA producers and the genetic basis of PHA synthesis capability. To achieve industrial-scale PHA production, efforts have been undertaken to discover new PHA-producing microbial strains with a larger catabolic versatility that can utilise inexpensive carbon sources (i.e., renewable agricultural or industrial wastes and by-products) [8]. The need for competent bacterial PHA producers has encouraged the search for highly productive PHA producers and the associated PHA-synthesising gene clusters through both traditional environmental isolation and modern metagenomic screening approaches, the latter allowing the remaining estimated 97% undiscovered bacteria to be exploited as well [5,9]. Among the PHA-synthesising gene clusters, the PHA synthase (*phaC*) gene is key to the catalysis of PHA formation from hydroxyacyl-CoA thioesters. Moreover, PhaC influences the final polymer composition as well as substrate specificity [10]. Yet, due to the uncultivability of most marine bacteria, only a limited fraction of PhaC enzymes have been identified, characterised, and expressed for these organisms [9].

The marine ecosystem is considered to still contain a large proportion of undiscovered natural products with various industrial applications [9]. Marine benthic organisms and associated habitats, such as sponges and sediments, are substrates for microbial growth, shelter, and environmental protection; thus, diverse microbial communities in these environments have been reported [11]. Sediment provides surface attachment for microbes, while marine sponges filter large volumes of water and accumulate microorganisms that pass the sponge’s defensive mechanisms. Due to the competitive microbial community and stressful living conditions in the wild caused by harsh environments, marine sponges and sediment contain complex microbial populations that produce rich secondary metabolites and natural products to compete for survival [11].

The harsh and competitive microenvironments in marine sponges and sediment are expected to contain metabolically versatile microbial communities, whereby one of the secondary metabolites that have been commonly extracted from marine bacteria is the PHA polymer [12]. In this study, microbiological and molecular-biological techniques were employed to characterise cultivable and uncultivable PHA-producing bacteria in the marine sediment and sponges (i.e., *Xestospongia muta* and *Aaptos aaptos*) of Redang and Bidong islands, located in the Malaysian region of the Coral Triangle.

## 2. Materials and Methods

### 2.1. Collection of Bacterial Samples

**Figure 1:**
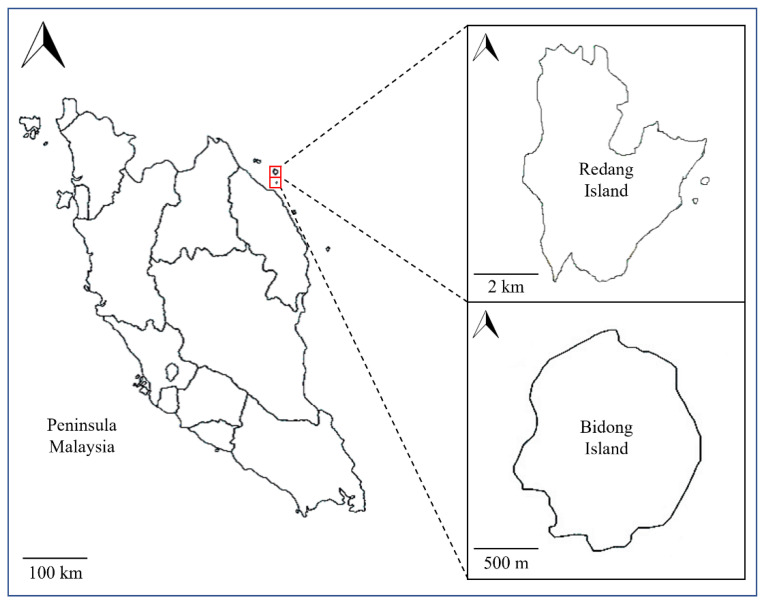
Map of Bidong Island and Redang Island, where marine sediment and sponges were collected.

Marine sponges and surficial coastal surface sediment were sampled from Bidong and Redang islands in Terengganu, Malaysia, within the Coral Triangle area (Figure 1) [13]. Grab was used to collect the surface sediment samples at 10 to 15 cm depth. Samples were immediately transferred to sterilised 250-mL Schott bottles, which were kept in an icebox until further analysis at the laboratory within 12 h of sample collection.

### 2.2. PHA Production and phaC Gene Identification in Sediment-Associated Bacterial Isolates with a Cultivation Approach

#### 2.2.1. Pre-Culture of Environmental Bacteria

First, marine sediment samples were suspended with 200 mL phosphate-buffered saline solution and prepared using 1.44 g Na_2_HPO_4_, 0.24 g KH_2_PO_4_, 8 g NaCl, and 0.2 g KCl. One mL each for the calibrated marine sediment samples and the coastal soil sample was added to 50 mL NR broth in a conical flask. The pre-culture was agitated at 180 rpm for 24 h at a temperature of 30 °C. An NR broth medium was made using 2 g yeast extract, 10 g peptone, and 10 g meat extract [14]. For the preparation of the NR agar, 14 g/L bacteriological agar powder (Oxoid, Dublin, Ireland) was included.

#### 2.2.2. Strain Isolation

Strains were isolated from the pre-cultures in conditions that induce PHA biosynthesis. More specifically, 1.5 mL of pre-culture was added to 50 mL mineral salts medium (MSM) (0.39 g/L of MgSO_4_·7H_2_O, 2.80 g/L KH_2_PO_4_, 3.32 g/L Na_2_HPO_4_, and 0.5 g/L NH_4_Cl) in a 250-mL flask, to which 20 g/L of a sole carbon source added was either 2.78 g/L glucose (HiMedia, Mumbai, India), 1.46 g/L sucrose (HiMedia, Mumbai, India), 5.54 g/L fructose (HiMedia, Mumbai, India), 0.79 g/L glycerol (Bendosen, Selangor, Malaysia), 0.79 g/L glycerine pitch, 1 g/L sweet water, or 4.47 g/L molasses, separately [15]. Glycerine pitch was obtained from Oleochemicals Industry, Penang, Malaysia [16]. Molasses and sweet water were obtained from the cane sugar refinery company Gula Padang Terap Sdn. Bhd. (14006-V), Kuala Nerang, Kedah, Malaysia. Prior to the addition of the pre-culture, 0.8 mL of a solution of trace elements was added to the medium (2.78 g/L FeSO_4_. 7H_2_O, 1.98 g/L MnCl_2_.2H_2_O, 1.67g/L CaCl_2_.2H_2_O, 2.81 g/L CoSO_4_.2H_2_O, 0.29 g/L ZnSO_4_.2H_2_O, and 0.17 g/L CuCl_2_.2H_2_O). The culture was agitated at 200 rpm and 30 °C, enabling growth in aerobic conditions. After 72 h, a tenfold dilution series was conducted, as described by Patel et al. [17]. The samples were then incubated on MSM agar for 48 to 72 h at 30 °C with higher carbon source concentrations (55.6 g/L glucose, 29.2 g/L sucrose, 55.4 g/L fructose, 15.8 g/L glycerol, 15.8 g/L glycerine pitch, or 20 g/L sweet water). Molasses-containing MSM agar was not required due to the growing absence in the MSM broth that used molasses as the sole carbon source. Following incubation, morphological observation was conducted to recognise single colonies of putative bacterial PHA producers according to distinct traits in terms of colour, shape, margin, and elevation. Strain isolation was performed by streak-purifying selected single colonies on fresh MSM agar plates with their respective carbon sources. The plates were then left to incubate for 72 h at 30 °C.

#### 2.2.3. Screening for PHA Presence

The plated isolates were confirmed for PHA presence by Nile blue screening. One mL of Nile blue dye, prepared by dissolving 0.1 mg Nile blue dye in 100 mL dimethyl sulfoxide (DMSO), was pipetted into each MSM plate for screening of PHA production as described [18]. The solution was slowly dripped at the plate edge to cover the whole colony surface. The plates were then analysed for fluorescence with the Spectroline Model CC-80 Ultraviolet Fluorescence Analysis Cabinet (Spectronics, New York, USA) under 365 nm ultraviolet (UV) light. Strains of colonies that displayed fluorescence were subjected to subsequent molecular identification analyses.

#### 2.2.4. PHA Production Using Shake-Flask Fermentation

After Nile blue screening, the three isolates were subjected to production and optimisation assessment under the selected range of parameters (C/N ratio, temperature, agitation speed, and MgSO_4_·7H_2_O and inoculum concentration) using a Nile red staining approach [19]. The sole carbon source used was the same as that used during the isolation stage in Section 2.2.2. The three strains that showed PHA production in a sole carbon source of glucose, sucrose, and glycerol at C/N ratios 30, 40, and 20, respectively, were cultivated in shake-flask fermentation, and their PHA production was analysed under selected ranges of temperature (30, 40, and 45 °C), MgSO_4_·7H_2_O concentration (1, 2, and 4 µM), agitation speed (100, 170, and 250 rpm), and inoculum concentration (1.5, 3.0, and 5.0% *v*/*v*). PHA production was analysed by measuring optical density at 660 nm (OD660). The upper temperature range for mesophilic bacteria (30–45 °C) was selected, which was suitable for PHA production processes in tropical regions without costly temperature control systems [20]. To prepare a pre-culture, two loops of bacterial culture were transferred from MSM agar plates that contained selected sole carbon sources into 25-mL flasks of NR broth, after which they were incubated at a temperature of 30 °C and an agitation of 180 rpm for 16 h. The culture was added to 50 mL MSM and other growth-supporting elements in 250 mL conical flasks for PHA biosynthesis. The nitrogen source used was NH_4_Cl with a fixed concentration of 0.5 g/L [19]. OD_660_ was measured at 24 h intervals. Rapid quantitative measurement of accumulated PHAs was performed by centrifuging 1 mL culture, followed by its resuspension in 40 μL Nile red and 1 mL distilled water, a 30-min room temperature incubation, and another centrifugation for 5 min at 12,000 rpm in a microcentrifuge. Next, 1 mL distilled water was added to the pellet, vortexed, and 150 μL of this solution was placed into a 96-well microplate for fluorescence emission and optical density measurement at 500-nm and 600-nm wavelengths, respectively, using the Synergy H1 microplate reader (Agilent, California, USA) and Gen5 (Version 3.03) software [21,22]. The following formula was applied in the C/N ratio calculation: C/N ratio = ([No. of carbon × molecular weight of carbon × *y* g/L]/Total molecular weight of carbon source)/([Number of nitrogen × molecular weight of nitrogen × *z* g/L]/Total molecular weight of nitrogen source), in which *y* is the concentration of carbon source used, and *z* is the concentration of nitrogen source used. Experiments were triplicated for each carbon source at a temperature, agitation, and duration of 30 °C, 170 rpm, and 72 h. After 72 h, the harvested culture was subjected to GC analysis and a graph was plotted to show the preliminary effects of temperature, MgSO_4_·7H_2_O concentration, agitation speed, and inoculum concentration against PHA production.

### 2.3. Gas Chromatography Analysis

After PHA production, the produced PHA was analysed using gas chromatography (GC), which was performed with Shimadzu GC-17A (Shimadzu, Kyoto, Japan) equipped with a fused silica capillary column, 30 m × 0.25 mm × 0.25 μm Supelco SPBTM-1 (Sigma-Aldrich, Missouri, USA), to chemically characterise the PHA produced by the isolated strains. The methanolysis process was conducted according to Braunegg et al. [23]; 15 mg lyophilised cells were exposed to the methanolysis solution, which consisted of CH_3_OH and H_2_SO_4_ at a ratio of 85:15 (% *v*/*v*). The mixture incubation was conducted at a temperature of 100 °C and for 2 h 20 min to convert the PHA monomers to methyl ester forms, after which the mixture cooled to 24 °C. Next, 1 mL of distilled water was inserted, followed by 30 s of vortexing. The bottom organic layer was transferred into a 7 mL screw-capped glass vial. Anhydrous sodium sulphate was added to the bijou bottle to eradicate the excess water. Approximately 0.5 mL of the resulting organic layer was shifted into a vial by ensuring that the sodium sulphate precipitate was opted out in the process. Later, 0.5 mL of the caprylic methyl (CME) solution was supplemented into the vial as an internal standard. A total of 2 µL of the vial mixture was injected into the GC for analysis.

### 2.4. Identification of PHA-Producing Strains from Sediment and Their PHA Synthases

After PHA production and GC analysis, the three isolates that were screened for a positive PHA presence were molecularly identified using 16S rRNA. Genomic DNA was extracted from the positive PHA producers using a Wizard^®^ Genomic DNA Purification Kit (Promega, Wisconsin, USA) according to the manufacturer’s protocol. Then, the 16S rRNA genes were amplified using the primers: 8F (5′-AGAGTTTGATCCTGGCTCAG-3′) and 1492R (5′-GGTTACCTTGTTACGACTT-3). The DNA sequencing analysis was processed at First Base Sdn Bhd, Kuala Lumpur, Malaysia. The query sequences were identified with their closest similarity on the Genbank NCBI database with the Basic Local Alignment Search Tool (BLAST) algorithm and SepsiTest. The Mr Bayes tool (Version 3.2.6) was applied to the phylogenetic relationship analysis between the genera, and they were assessed using the Bayesian inference strategy using 10,000,000 generations with 1000 burn-in, GTR+G+I model of the DNA substitution for the concatenated dataset. Multiple sequence alignment for all the obtained genome sequences and other known PHA producers was first performed with the online Multiple Alignment using Fast Fourier Transform (MAFFT) (Version 7.397) tool. The 16S rRNA sequences of the PHA-producing isolates, *Sphingobacterium mizutaii* UMTKB-6, *Alcaligenes faecalis* UMTKB-7, and *Acinetobacter calcoaceticus* UMTKB-8, have been published in GenBank under the accession numbers of OP256902, OP256903, and OP256904, respectively.

Next, the PHA synthases of the isolates were isolated and identified. Based on phylogenetic determination of the isolated strains, the corresponding *phaC* nucleotide sequences were imported from National Center for Biotechnology Information (NCBI) and Uniprot databases. A protein-based indirect speculation method of putative nucleic acid sequences was applied for designing the primers for the isolate, *Sphingobacterium mizutaii*, for which the *phaC* genes have not been previously characterised. SnapGene software (Version 4.3.7) was used to design the primers (Table 1). According to conserved regions, PhaC enzymes are categorised into several classes [24]. Using NCBI protein and BLAST tools, the *Sphingobacterium mizutaii* genome sequence was aligned against the amino acid sequences of all four synthase classes (Class I, II, III, and VI). Subsequently, the resulting uncharacterised and hypothetical protein sequences were used to construct a multiple sequence alignment with PhaC amino acid sequences of different classes employing Clustal Omega. According to the multiple sequence alignment, retrieved proteins were divided into two groups (I and II), which displayed conserved regions with Class I and Class III PhaC, respectively. The corresponding nucleotide sequences of these hypothetical proteins were aligned to query the conserved regions for primer design use (Table 1).

### 2.5. phaC Gene Identification and Screening of phaC Taxa in Sponge-Associated Bacterial Metagenomes without Cultivation Approach

#### 2.5.1. Extraction of Bacterial Metagenome

In order to also rapidly screen for the putative presence of uncultivable PHA-producing bacteria, sponge-associated bacterial metagenome was extracted and characterised. First, sponge tissue was submerged and lightly shaken in distilled water with sterile forceps to detach external bacteria. The sponge pinacoderm (outermost layer) was cut off using sterile forceps and a scalpel. The sponge tissue was diced into 3-mm^3^ small pieces with a sterile scalpel and then ground using liquid nitrogen with a pestle and mortar until the liquid nitrogen evaporated. The metagenome from the mentioned sponge tissue was extracted with phenol-chloroform isoamyl alcohol (PCI), as described by Beloqui et al. [25]. The metagenome purity and concentration were measured with a Nanodrop™ 2000 Spectrophotometer (Thermo Fisher Scientific, Waltham, MA, USA).

#### 2.5.2. Bacterial Diversity Profiling Using 16S rRNA Amplicon Sequencing Analysis

The sponge-associated bacterial diversity was also profiled to preliminarily screen for putative PHA-producing taxa. After the extraction of the sponge-associated bacterial metagenome, the V3 and V4 regions of the 16S rRNA genes of the sponge bacterial metagenomes were amplified using Phusion^®^ High-Fidelity PCR Master Mix (New England Biolabs, Ipswich, MA, USA) based on the manufacturer’s instructions with the specific primers 341F and 806R (Table S3) [26]. Post-agarose gel electrophoresis and UV gel viewing, intense bands from 400 bp to 450 bp were purified with Qiagen Gel Extraction Kit (Qiagen, Hilden, Germany) and subjected to sequencing library construction with NEBNext^®^ Ultra™ DNA Library Pre Kit (New England Biolabs) for Illumina according to the manufacturer’s instructions, followed by adding index codes. The concentration and purity of the library were quality checked with Qubit^®^ 2.0 Fluorometer (Thermo Fisher Scientific) and the Bioanalyzer 2100 system (Agilent), respectively. Library sequencing and construction of 250-bp paired-end reads were performed with an Illumina platform HiSeq2500 Rapid Mode PE-250.

The paired-end reads were demultiplexed according to their unique barcode, shortened through deletion of primer sequence and barcode, and then combined using the Fast Length Adjustment of Short Reads software (FLASH Version 1.2.7) to produce raw tags. Then, the Quantitative Insights into Microbial Ecology software (QIIME Version 1.7.0) was applied to filter the raw tags into clean tags. Subsequently, the UCHIME algorithm and ChimeraSlayer reference database were applied to eliminate chimaera sequences to produce effective tags. Afterwards, the Uparse software (Version 7.0.1001) was used to analyse the effective tags into equal or above 97% similarity, which was appointed as the same operational taxonomic unit (OTU). The representative sequence of individual OTUs was further annotated and classified with the Ribosomal Database Project (RDP) classifier (Version 2.2) and GreenGene Database. The Multiple Sequence Comparison by Log-Expectation (MUSCLE) software (Version 3.8.31) was used to conduct multiple sequence alignments. The OTU annotations were visually presented in evolutionary trees, abundance heatmaps, as well as abundance pie and bar charts. GenBank accession numbers from MT464469 to MT465036 represent the partial 16S rRNA genes of the detected OTUs. The public repository Discover Mendeley Data was used to publish other analysed and raw data (http://doi.org/10.17632/zrcks5s8xp, accessed on 15 October 2022) [15].

#### 2.5.3. Identification of PHA Synthases from Marine Sponge Bacterial Metagenome

The sponge-associated bacterial metagenome was screened for PHA synthases using polymerase chain reaction (PCR) amplification, which was performed with EconoTaq^®^ PLUS 2X Master Mix (Lucigen, Teddington, UK) based on the manufacturer’s instructions with the following cycle: pre-denaturation for 3 min at 95 °C, denaturation for 30 s at 95 °C, annealing for 1 min at 56 °C, extension for 90 s at 72 °C, and final extension for 5 min at 72 °C with Applied Biosystems™ Veriti 96-Well Thermal Cycler (Thermo Fisher Scientific) [27]. For the amplification of Class I and II *phaC* genes, forward primer CF1 (5′-ATCAACAA(A/G)T(A/T)CTAC(A/G)TC(C/T)T(C/G)GACCT-3′), and reverse primer CR4 (5′-AGGTAGTTGT(C/T)GAC(C/G)(A/C)(A/C)(A/G)TAG(G/T)TCCA-3′) were used [27]. Next, forward primer CF2 (5′-GT(C/G)TTC(A/G)T(C/G)(A/G)T(C/G)(A/T)(C/G)CTGGCGCAACCC-3′) and reverse primer CR4 were used in semi-nested PCR with a similar protocol as described above.

Agarose gel electrophoresis [28] and gel visualisation were performed with PowerPac™ Basic power supply (Bio-Rad Laboratories, CA, USA) and Gel Doc™ EZ Imager (Bio-Rad Laboratories), respectively. DNA sequencing analysis was performed at First BASE Laboratories Sdn Bhd using Applied Biosystems™ Genetic Analyzer (Thermo Fisher Scientific) with Sanger sequencing. Subsequently, query sequences were align-edited with BioEdit software (Version 7.2.6) and then identified to their closest similarities with the NCBI sequence database using the BLAST tool. Sequences have been published in the GenBank database with accession numbers MF457754, MF457753, and MF437016.

## 3. Results and Discussion

### 3.1. Identification of PHA-Producing Strains from Sediment

**Figure 2:**
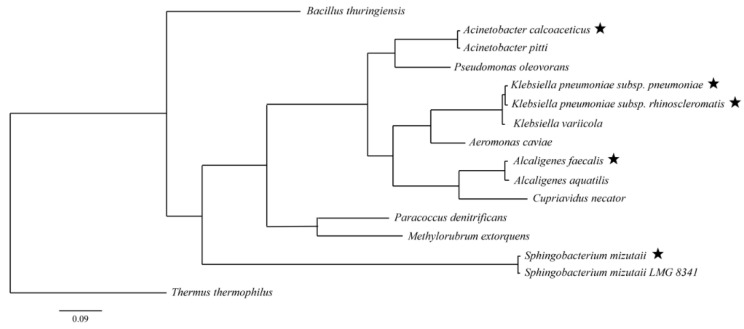
Phylogenetic tree constructed using Bayesian inference strategy and fig tree demonstrating the relations between the isolated strains (★) and other related species.

A total of 49 isolates were selected for further PHA screening after visual observation. PHA-producing strains were observed as fluorescent under UV radiation as a result of the existence of PHA granules in the cell. A total of 17 fluorescent-positive isolates post-PHA screening were subjected to PHA biosynthesis on all sole carbon sources since PHA dyed by Nile blue released fluorescence [29]. The three strains with the most intense fluorescence were picked for subsequent gas chromatography analysis, which were *Sphingobacterium mizutaii* UMTKB-6 cultured in sucrose, *Alcaligenes faecalis* UMTKB-7 cultured in glucose, and *Acinetobacter calcoaceticus* UMTKB-8 cultured in glycerol as sole carbon sources. The other two unselected strains from the species *Klebsiella pneumoniae* were excluded due to pathogenicity. Molecular identification of species with DNA sequencing and BLAST analysis confirmed the three strains as *Sphingobacterium mizutaii* (strain similarity 99.0%), *Acinetobacter calcoaceticus* (strain similarity 99.3%), and *Alcaligenes faecalis* (strain similarity 99.0%). The phylogenetic relations between the strains were assessed using the Bayesian strategy (Figure 2). The *S. mizutaii* strain in this study, from the phylum Bacteroidetes, revealed previously unreported PHA-producing ability. *A. calcoaceticus* and *A. faecalis* which have been previously characterised for PHA production, belong to the same phylum Proteobacteria [30,31]. Of these five sediment-isolated PHA-producing strains, only the genus *Acinetobacter* was detected in the sponge-associated bacterial communities investigated in this study. However, it is known as a common environmental bacterial genus that is also found in water and soil [32]. A comprehensive search across multiple environments is thus still needed to exploit competent PHA producers from strain and gene mining research efforts.

### 3.2. Production and Characterisation of PHA from Isolated Strains

**Figure 3:**
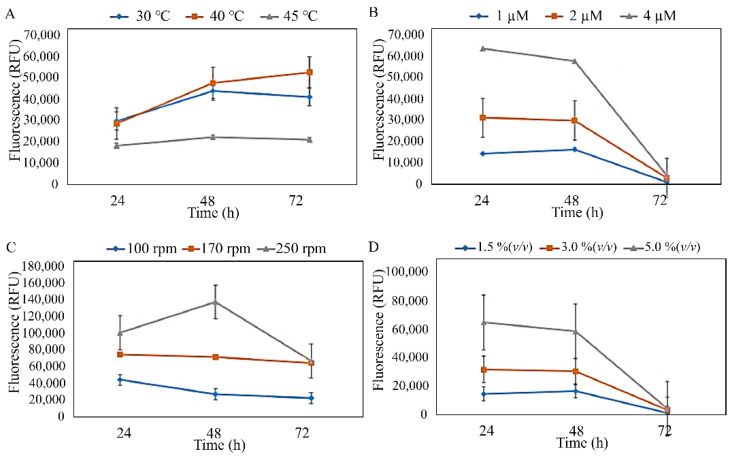
Identification of suitable culture parameters for PHA biosynthesis by *S. mizutaii* UMTKB-6 cultured in sucrose. (**A**). Effect of temperature on PHA production, (**B**). effect of MgSO_4_·7H_2_O concentration on PHA production, (**C**). effect of agitation on PHA production, (**D**). effect of inoculum concentration on PHA.

**Figure 4:**
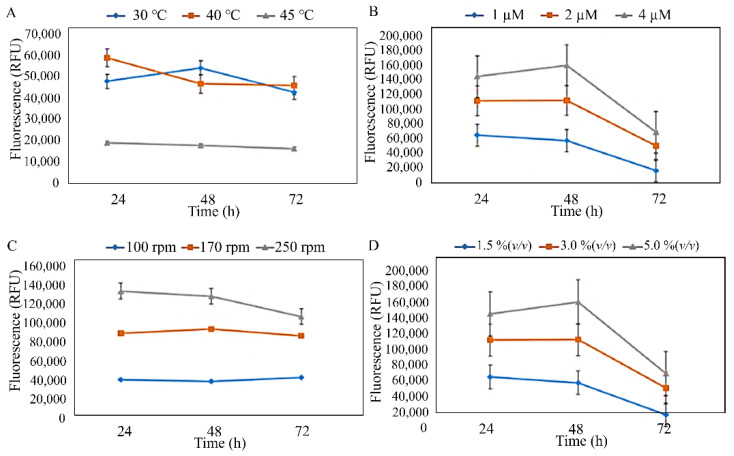
Identification of suitable culture parameters for PHA biosynthesis by *A. faecalis* UMTKB-7 cultured in glucose. (**A**). Effect of temperature on PHA production, (**B**). effect of MgSO_4_·7H_2_O concentration on PHA production, (**C**). effect of agitation on PHA production, (**D**). effect of inoculum concentration on PHA.

**Figure 5:**
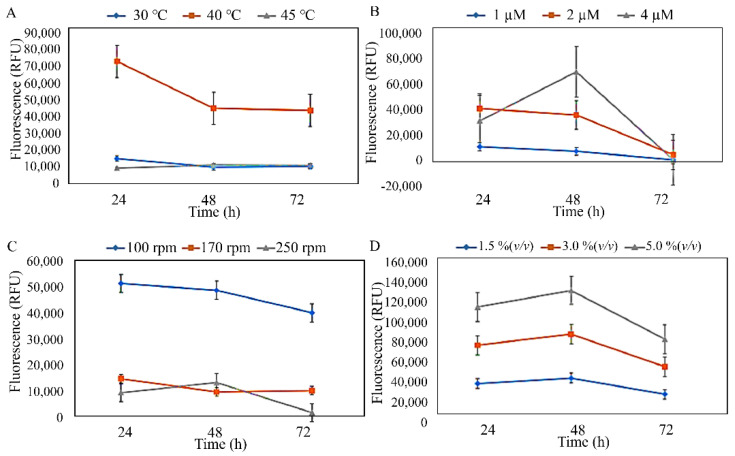
Identification of suitable culture parameters for PHA biosynthesis by *A. calcoaceticus* UMTKB-8 cultured in glycerol. (**A**). Effect of temperature on PHA production, (**B**). effect of MgSO_4_·7H_2_O concentration on PHA production, (**C**). effect of agitation on PHA production, (**D**). effect of inoculum concentration on PHA production.

The three isolates were subjected to preliminary optimisation, which was conducted to estimate the effects of temperature, MgSO_4_·7H_2_O concentration, agitation speed, and inoculum concentration against the PHA production of the isolates (Figure 3, Figure 4 and Figure 5). The preliminary investigation revealed that the temperature, MgSO_4_·7H_2_O concentration, agitation speed, and inoculum concentration of 40 °C, 1 µM, 100–170 rpm, and 1.5–3.0% (*v*/*v*), respectively, were generally preferred by the isolated strains. However, *S. mizutaii* UMTKB-6 showed a preference for 1.5% (*v*/*v*) of inoculum concentration. All strains showed a mixed preference for agitation speeds between 100 and 170 rpm but altogether less preference for the 250-rpm agitation speed.

The PHA produced from the three selected isolates were also subjected to GC chromatography for compositional analysis of the produced PHAs. Based on GC data, the monomers produced by the isolated strains were detected as hydroxyvalerate (HV), hydroxydecanoate (HD), hydroxyunidecanoate (HUD), hydroxyoctanoate (HO), and hydroxyhexanoate (HHx). The monomer HO was detected when molasses was utilised as the sole carbon source (data not shown); however, only trace amounts of PHAs were produced in this case.

The Gram-negative isolate, *Acinetobacter* *calcoaceticus* UMTKB-8, was shown to produce PHA using glycerol as the sole carbon source. GC analysis revealed that the produced PHAs consisted of HV, HD, and HUD monomers when *A. calcoaceticus* utilised glycerine pitch as the sole carbon source at a C/N ratio of 20 (Table 2). *Acinetobacter sp.* was previously reported to utilise glycerol for the production of MCL and SCL PHAs consisting of 9 mol% 3-hydroxytetradecanoate (3HTD), 52 mol% 3-hydroxydodecanoate (3HDD), and 39 mol% 3HD [30]. This study reported the ability of a related strain to produce 3HV monomers without the presence of precursors [30,33]. Previously, other bacterial species were reported to biosynthesise copolyester without adding precursors, such as *Bacillus licheniformis* and *Hydrogenophaga pseudoflava* [33]. According to Muangwong et al., the occurrence of unsaturated and saturated fatty acids in PHA could be attributable to a probable linkage between the de novo fatty acid and β-oxidation biosynthesis pathways [34].

*Alcaligenesfaecalis* UMTKB-7 was found to produce PHAs consisting of HD and HUD monomers using glucose as the sole carbon source at a C/N ratio of 30. Although most studies reported the PHA-depolymerising ability of *A. faecalis*, a recent study on different subspecies of *A. faecalis* showed that the genome of *A. faecalis* subsp. *phenolicus* encoded enzymes for PHA synthesis [31]. In general, the *Alcaligenes* genus has only been reported to produce SCL PHA monomers, and scarce data are available on MCL PHA monomer production, which could be due to the low efficiency in utilising its carbon source [34,35]. As is the case for other bacteria, glucose was the preferred carbon source of PHA production for different *Alcaligenes* species [35].

Biosynthesis of PHA composed of dissimilar kinds of MCL PHA monomers was observed for *Sphingobacterium mizutaii* UMTKB-6 upon growing the strain with sucrose as the sole carbon source at a C/N ratio of 40, namely HO, HD, HUD, and HHx. *S. mizutaii* has not been described previously for PHA production. Not all PHA producers can utilise sucrose, which is a relatively inexpensive and commonly available sugar, as a carbon source for PHA production. For example, *Cupriavidus necator*, the most common PHA-producing bacterium, lacks the capacity to utilise sucrose due to the absence of sucrose hydrolase gene [36]. This advantage endows *S. mizutaii* with great potential for the industrial production of PHA in many applications. To obtain sufficiently high DCW and PHA content with the necessary molar fractions, it is crucial to employ optimal concentrations of precursors and carbon sources.

### 3.3. Identification of phaC from Sediment

The next objective aimed to identify the sequences of three *phaC* genes from cultivated sediment isolates, which were *A. calcoaceticus*, *S. mizutaii*, and *A. faecalis* (Table 3). Amplification of *phaC* was obtained with the use of specific primers targeting Class I *phaC*. *A. calcoaceticus* and *A. faecalis* were expected to harbour Class I *phaC* similar to other PHA-producing species within the same genera of *Alcaligenes* and *Acinetobacter*. However, the production of PHA by *S. mizutaii* was not described in previous literature. Multiple sequence alignments of the hypothetical proteins of *S. mizutaii* and the *phaC* sequences of Class I, II, and III (obtained from the NCBI GenBank database) indicated that the hypothetical sequences exhibited more conserved residues with Class I and III *phaC*.

The substrate specificity of *phaC*, available carbon sources, and metabolic pathways can affect the composition and type of PHA biosynthesised [37]. This study allowed the discovery of potential strains with *phaC*, which can polymerise different PHAs for a higher yield through the manipulation of the type and quantity of carbon sources. Furthermore, the presence of Class I-like PHA synthases from the isolated strains that exhibited previously unreported production of MCL PHA indicated the possible presence of additional PHA synthases from Class II, III or IV. Given that the strains isolated in this study were only analysed using Class I-specific primers hence the screening for additional presence of other PHA synthases in the strains is highly recommended.

### 3.4. Bacterial Diversity Profiling in Marine Sponges

**Figure 6:**
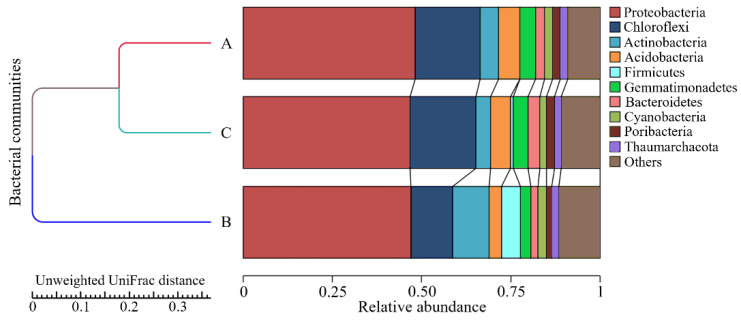
A relative abundance stacked bar chart of the top ten dominant phyla detected in the sponge-associated bacterial diversity profiles of this study. A, *A. aaptos* prokaryotic community of Bidong Island; B, *A. aaptos* prokaryotic community of Redang Island; C, *X. muta* prokaryotic community of Bidong Island.

The sponge-associated bacterial diversity profiling revealed the occurrence of at least 10 bacterial phyla within *A. aaptos* (Figure 6). Interestingly, the *A. aaptos* community composition was similarly diverse and abundant as the high-microbial-abundant (HMA) *X. muta*, suggesting that *A. aaptos* is an HMA marine sponge [38]. Low-microbial-abundant (LMA) sponges usually harbour only one to two phyla, while HMA sponges harbour at least eight phyla [39]. Another sponge of the same genus, *A. suberitoides*, was also reported as an HMA sponge [40]. In addition, HMA sponges generally have narrower canals, which coincide with the physical characteristic of *A. aaptos* [41]. For the *X. muta* host sponge, a comparison with past reports showed that the major phyla of *X. muta* in the Malaysian region of the South China Sea were roughly similar to *X. muta* in the Caribbean Sea [42,43]. The major phyla in the *X. muta* bacterial community from Bidong Island were Actinobacteria, Acidobacteria, Chloroflexi, Cyanobacteria, Proteobacteria, Gemmatimonadetes, and Nitrospirae, whereas the major phyla of geographically distant *X. muta* from the Caribbean were Actinobacteria, Acidobacteria, Chloroflexi, Cyanobacteria, Proteobacteria, Poribacteria, and an Archaean phylum, Thaumarchaeota [44,45]. However, a larger number of samples across a broader temporal and spatial range is required to accumulate more evidence for the confirmation of the microbial abundance in *A. aaptos*.

Overall, the findings showed the detection of diverse bacterial taxa that implied the presence of various potential biotechnological abilities and metabolites in marine sponges besides PHA production, such as antibacterial activity, biodegradation, and demethylation [46,47,48]. The dominant bacterial families from the marine sponges were Burkholderiaceae, Comamonadaceae, Desulfurellaceae, Nitrospiraceae, Oxalobacteraceae, and Rhodobacteraceae. These ecologically diverse families comprised chemoorganotrophs, chemolithotrophs, photoheterotrophs, and methylotrophs, among which were bacteria with nutrient-cycling roles, explicitly nitrogen-fixing and sulphur-reducing bacteria [49]. The role of marine sponges as filter feeders provides a nutrient- and pollutant-rich internal environment for diverse bacteria [50]. Therefore, PHA-synthesising abilities can be present among the bacteria associated with marine sponges due to the need for survival in unfavourable conditions [8]. Some PHA-producing bacterial taxa were detected in the marine sponge-associated bacterial metagenome.

### 3.5. PHA-Producing Taxa Detected in Sponge-Associated Bacteria

**Figure 7:**
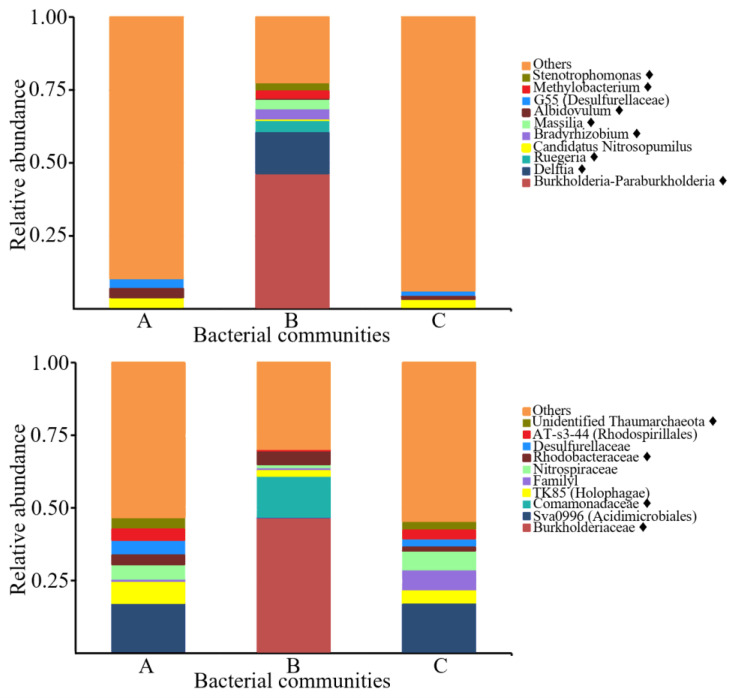
A relative abundance stacked bar chart of the top ten dominant genera (top) and families (bottom) detected in the sponge-associated bacterial diversity profiles of this study. A, *A. aaptos* prokaryotic community of Bidong Island; B, *A. aaptos* prokaryotic community of Redang Island; C, *X. muta* prokaryotic community of Bidong Island. Taxa that consist of PHA producers are denoted by a solid-filled diamond symbol (♦).

The marine sponge-associated bacterial diversity profiling revealed that eight out of the ten most dominant genera and four out of the ten most dominant families were previously published PHA producers (Figure 7) [51,52]. However, not all species classified as PHA-producing genera are experimentally shown to produce PHA. A general mapping of PHA-producing genera in an environmental sample may only imply the existence of a broad range of PHA producers that further need to be characterised. The marine ecosystem likely comprises novel unexploited gene pools, and more bacterial PHA producers are expected to be revealed in the future from *phaC* gene mining efforts in marine sponges.

The distinct environmental conditions in marine sponges (e.g., anaerobic regions) place bacterial populations in unfavourable living environments, such as conditions with low availability of electron acceptors, oxygen, essential nutrients, space, light, and other growth factors [53]. To compete for survival in inconsistent living conditions, some bacteria utilised alternative pathways to synthesise and accumulate PHA as an alternative energy source [50]. Hence, this study and previously existing reports suggest that PHA-synthesising bacteria can be present in environmentally stressed marine sponges, thus triggering PHA-producing bacteria to adapt and functionally stabilise their living mechanisms towards unfavourable environments [54]. Together with the previously described PHA-producing taxa in the investigated marine sponge bacterial metagenomes, the diverse sponge-associated bacterial communities and the recognition of marine sponges as bacterial hotspots strongly suggest sponges as a future source of bacteria and genetic pools for the gene mining of PHA synthases and producers. Future investigations on bacteria from marine sponges, sediment, and other microbial hotspots, could disclose future PHA producers, *phaC* and their gene clusters for industrial and medical exploitation.

### 3.6. Identification of phaC from Marine Sponges

The diversity profiling of marine sponge-associated bacterial metagenome indicated that the presence of PHA producers was highly possible due to the detection of PHA-producing taxa among the top dominant genera and families (Figure 6). As a result, the marine sponge-associated bacterial metagenome was further screened for the presence of the *phaC* gene via PCR amplification using *phaC-specific* primers.

For the extraction of *phaC* genes from the sponge-associated bacterial metagenomes, clones 2, 1B, and 2B were identified as *Pseudomonas stutzeri* 1317 *phaC* (Class II), uncultured bacterium AR5-9d_16 *phaC* (Class III), and *Rhodocista pekingensis phaC* (Class I), with an identical similarity of 88.0%, 93.0%, and 74.0%, respectively (Table 4) [55]. No Class IV *phaC* was detected in this study. The discovery of *phaC* from Class I, II, and III, with mostly Class I *phaC*, was expected since Class I *phaC* are known to be more commonly detected [55]. However, also, in this case, a bias towards mostly detecting Class I *phaC* genes may also be due to limitations in primer design. A future improved design of primers for other classes of *phaC* could contribute to the discovery of new *phaC* genes.

## 4. Conclusions

The sediment isolate, *A. calcoaceticus*, produces copolymeric PHA without the addition of precursors and separately uses glycerine pitch and glycerol as renewable and economical carbon sources. The production of MCL PHA monomers using sucrose was detected in another sediment isolate, *S. mizutaii*, that has not before been reported to produce PHA. Also, an *A. faecalis* strain was isolated that produces PHA with glucose as the sole carbon source. For all three isolates, phaC genes have been identified and characterised as Class I. From the sponge-associated metagenome, three *phaC* genes similar to Class I, II, and III categories, were identified as *Rhodocista pekingensis phaC*, *Pseudomonas stutzeri* 1317 *phaC*, and uncultured bacterium AR5-9d_16 *phaC*, respectively.

The abundance of marine PHA-producing taxa may be comparable to that of their terrestrial counterpart. It is also apparent from the cultivation and non-cultivation approaches used in this study that strain fermentation and metabolite biosynthesis are achievable with the former, while gene manipulation and subsequent downstream biosynthesis are the focuses of the latter method. Extended research that could answer the research questions and limitations posed by this study include (1) metagenomic *phaC* or 16S rRNA amplicon sequencing on the sediment- or sponge-associated microbial communities, (2) microbial diversity profiling on the sediment- or sponge-associated microbial community over extended spatial and temporal range, and (3) the gene mining and bioengineering of *phaC* and PHA biosynthetic gene clusters from marine bacterial metagenomes to uncover super PHA producers.

## Figures and Tables

**Table 1:** Primers for *phaC* amplification in isolated strains. Primers for *phaC* amplification in *Sphingobacterium mizutaii* were designed by protein-based indirect speculation of the possible nucleic acid sequences method.

Strain	Primer Sequence (5′-3′)	Database Reference
*Acinetobacter Calcoaceticus*	* F: 5-ATGCTTAGTATTGAATTTTT-3	UniProt
	R: 5-TTATGGCTCATCCTCACG-3	(EMBL:SUU05450.1)
*Alcaligenes Faecalis*	F: 5′-ATGACTTCTGAATCATCC-3	NCBI Protein
	R: 5′-TTAACTGGCGCGCACTTTCA-3	(NZ_CP013119.1)
**Primer pair**	**Group**	**Primer sequence (5′-3′)**
1	I	* F: ATGAGAAAATACCTAATCCT
		R: TTATCGATTTACGTTCTG
2	I	F: ATGCATTTTATACTCATTTC
		R: TCACAGCAATGCAATTAATAAAACT
3	II	F: ATGATCTATTCACTAACAAG
		R: TTAATTTTTTACAATATACTCCTTAA
4	II	F: GTGTCAAAAAACCATAAAGGAAAT
		R: CTAGTGGAATCGATCTGG

* F, forward primer; R, reverse primer.

**Table 2:** PHA content and monomer composition of PHA biosynthesised by *Sphingobacterium mizutaii* UMTKB-6, *Alcaligenes faecalis* UMTKB-7, and *Acinetobacter calcoaceticus* UMTKB-8.

Strain	Carbon Source	PHA Content (wt%)	Monomer Composition (mol%)				
			3HV	3HO	3HD	3HUD	3HHx
*Sphingobacterium mizutaii*	Sucrose	37.4	-	15	28	49	8
UMTKB-6							
*Alcaligenes faecalis*	Glucose	61.1	Tr	-	25	74	-
UMTKB-7							
*Acinetobacter calcoaceticus*	Glycerol	49.4	Tr	-	Tr	99	-
UMTKB-8							

- no monomers detected. Tr trace amount detected.

**Table 3:** PHA synthase (*phaC*) genes identified from strain isolates from sediment-associated bacteria.

Strain Isolates from Sediment-Associated Bacterial Culture			
Strain Isolate	Closest Species	*phaC* Class	Strain Identity (%)
UMTKB-6	*Sphingobacterium mizutaii*	I	99.0
UMTKB-7	*Alcaligenes faecalis*	I	99.0
UMTKB-8	*Acinetobacter calcoaceticus*	I	99.3

**Table 4:** PHA synthase (*phaC*) genes identified from strain isolates from marine sponge-associated metagenome.

Cloned Genes from Sponge-Associated Bacterial Metagenome			
Gene Clone	Closest *phaC* Gene	*phaC* Class	Gene Identity (%)
2	*Pseudomonas stutzeri* 1317 *phaC* (AY278219.1)	II	88.0
1B	Uncultured bacterium AR5-9d_16 *phaC* (AB220790.1)	III	93.0
2B	*Rhodocista pekingensis phaC* (AY283802.1)	I	74.0

## Data Availability

The data presented in this study are openly available in Discover Mendeley Data at doi:10.17632/zrcks5s8xp. This data can be found here: http://doi.org/10.17632/zrcks5s8xp, accessed on 15 October 2022.

## Supplementary Materials

The following supporting information can be downloaded at: https://www.mdpi.com/article/10.3390/microorganisms10102057/s1, Figure S1: The roles and functions of metagenome extraction and gene extraction in the downstream applications and future study of the isolated genes.; Figure S2: The roles of marine sponges, sediment, and their bacterial symbionts in the advances of marine natural products within the fields of biochemistry and molecular biology. Figure S3: Workflow schematics from screening for PHA-producing strains using Nile blue, PHA production using shaken-flask fermentation, to PHA composition analysis using gas chromatography; Figure S4: Donut chart showing the relative abundance of classes detected in the bacterial diversity profiles of this study and another similar study accessible via the MG-RAST public repository for comparison [42,43]. Starting from the inner circle are the bacterial communities from (1) *A. aaptos* of Redang Is-land; (2) *X. muta* of Bidong Island; (3) *A. aaptos* of Bidong Island; (4) *A. corrugata* of Broward County; (5) *A. corrugata* of Fort Lauderdale; (6) *A. corrugata* of Summerland Key; Text S1: 16S rRNA sequence of Sphingobacterium mizutaii strain UMTKB-6; Text S2: 16S rRNA sequence of *Alcaligenes faecalis* subsp. *faecalis* strain UMTKB-7; Text S3: 16S rRNA sequence of *Acinetobacter calcoaceticus* strain UMTKB-8; Table S1: Retention times and peak areas of the detected monomers for *Sphingobacterium mizutaii* UMTKB-6, *Alcaligenes faecalis* UMTKB-7, and *Acinetobacter calcoaceticus* UMTKB-8; Table S2: Cell dry weight of *Sphingobacterium mizutaii* UMTKB-6, *Alcaligenes faecalis* UMTKB-7, and *Acinetobacter calcoaceticus* UMTKB-8.; Table S3: Primers 341F and 806R targeting 16S rRNA genes of regions V3 and V4 [26].
