# Supplementary material for: Polyhydroxyalkanoate (PHA) Biopolymer Synthesis by Marine Bacteria of the Malaysian Coral Triangle Region and Mining for PHA Synthase Genes
Source: Microorganisms. 2022 Oct 18;10(10):2057. doi: 10.3390/microorganisms10102057 (PMC9607975; doi:10.3390/microorganisms10102057)
Supplement: Supplementary file 1 [file microorganisms-10-02057-s001.zip › microorganisms-1911667-supplementary.pdf]

Supplementary Material

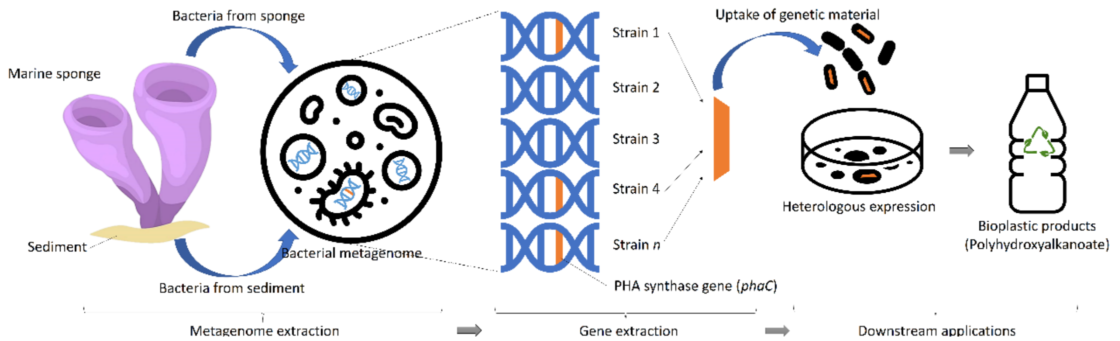

Figure S1. The roles and functions of metagenome extraction and gene extraction in the downstream applications and future study of the isolated genes.

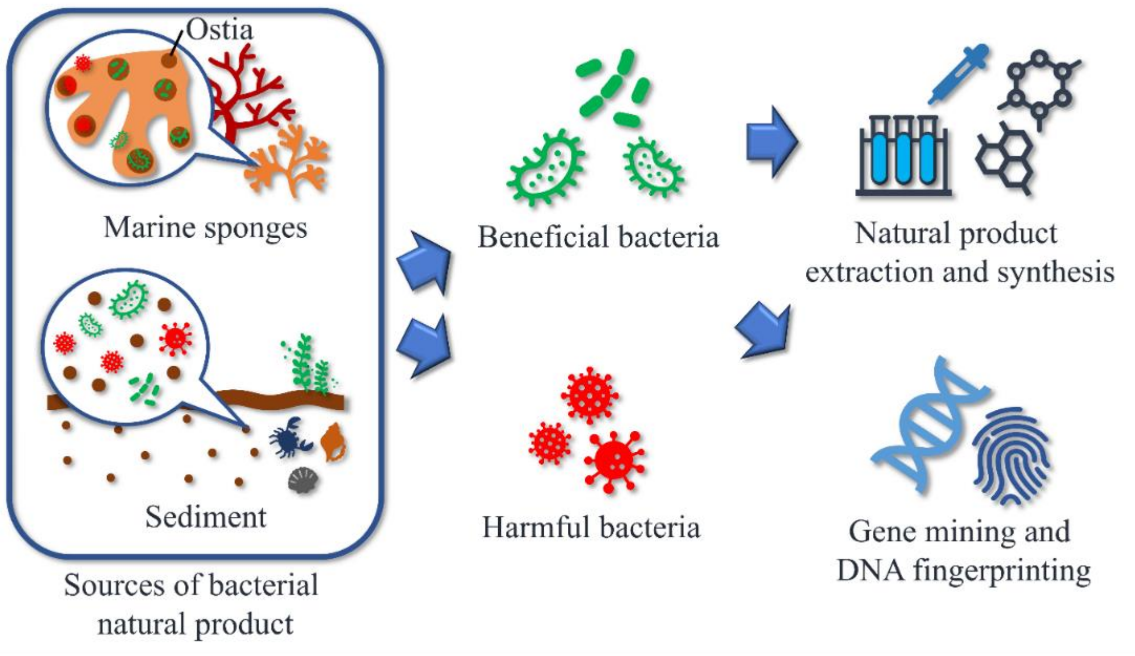

Figure S2. The roles of marine sponges, sediment, and their bacterial symbionts in the advances of marine natural products within the fields of biochemistry and molecular biology.

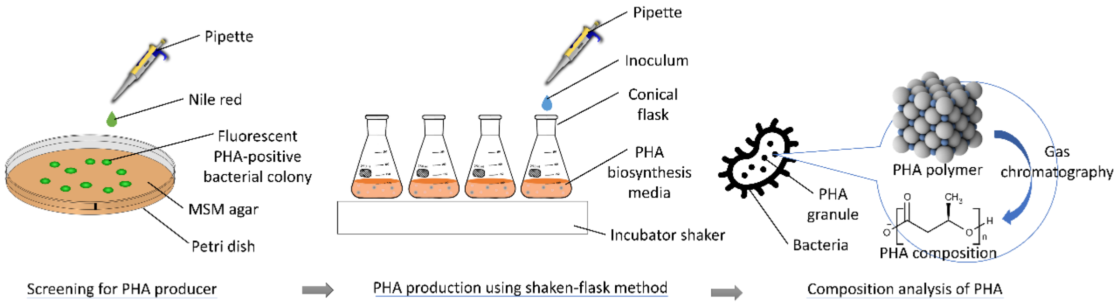

Figure S3. Workflow schematics from screening for PHA-producing strains using Nile blue, PHA production using shaken-flask fermentation, to PHA composition analysis using gas chromatography.

Text S1. 16S rRNA sequence of *Sphingobacterium mizutaii* strain UMTKB-6
CCACGCTCCTTGCGGTTACATGCTTTAGGTACCCCCAGCTTTCATGGCTTGACGGGCGGTGTGT
ACAAGGCCCGGGAACGTATTCACCGCGTCATTGCTGATACGCGATTACTAGCGAATCCAACT
TCACGGGGTCGAGTTGCAGACCCCGATCCGAACTGTGAATGGCTTTTCGAGATTGGCACCAC
ATTGCTGTGTAGCTGCCCGCTGTACCATCCATTGTAGCACGTGTGTAGCCCCGGACGTAAGGG
CCATGATGACTTGACGTCGTCCCCGCCTTCCTCTCTGCTTGCGCAGGCAGTCTGTTTAGAGTCC
CCACCTTGACGTGCTGGCAACTAAACATAGGGGTTGCGCTCGTTGCGGGACTTAACCCAACA
CCTCACGGCACGAGCTGACGACAGCCATGCAGCACCTAGTTTCCTGTCCCGAAGGACGGGCG
CGTCTCTGCGCCCTTCAGTAACTTTCAAGCCCGGGTAAGGTTCCTCGCGTATCATCGAATTAA
ACCACATGCTCCTCCGCTTGTGCGGGCCCCCGTCAATTCCTTTGAGTTTCACCCTTGCGGGCGT
ACTCCCCAGGTGGATGACTTAACGCTTTCGCTTGGACGCTTACGGTATATCGCAAACATCGAG
TCATCATCGTTTAGGGCGTGGACTACCAGGGTATCTAATCCTGTTCGATCCCCACGCTTTCGTG
CATCAGCGTCAATAACGGCTTAGACAGCTGCCTTCGCAATCGGTGTTCTGAGACATATCTATG
CATTTCACCGCTACTTGTCTCATTCCGCCGTCTTCAACCGCATTCAAGCACTTCAGTATCAAGG
GCACTGCGACAGTTGAGCTGCCGTCTTTCACCCCTGACTTAAAGTGCCGCCTACGCACCCTTT
AAACCCAATAGATCCGGATAACGCTCGGATCCTCCGTATTACCGCGGCTGCTGGCACGGAGT
TAGCCGATCCTTATTCTTTAGGTACATTCAGCCCGCTACACGTAGCGGGGTTTATTCCCTAACA
AAAGCAGTTTACAACCCATAGGCAGTCGTCCTGCACGCGGCATGGCTGGTTCAGGGTTGCCC
CCATTGACCATATTCCTTACTGCTGCCTCCCGTAGGAATCTGGTCCGTGTCTCAGTACCATGTG
GGGGATTCCCTCTCAATCCCCTAGCATCGTCGCCTTGGTGGGGCGTTACCCCACCACTAGCTA
ATGTCGAGCCATTCCACCCAAAAATTGACTACCTCCCGATGCCGGAAAATGCCAGGGGGGGT
TAATCCGGATTTCCCCGGCCCCCCAAATGGGGGTGCCCGTTTACCCCCCCGGGGCCCCCCCCT
CGGGGACCTACCAAGGAACCCCCCCAAATT

Text S2. 16S rRNA sequence of *Alcaligenes faecalis* subsp. *faecalis* strain UMTKB-7
GCTTTACATGCAGTCGAACGGCAGCGCGAGAGAGCTTGCTCTCTTGGCGGCGAGTGGCGGAC
GGGTGAGTAATATATCGGAACGTGCCCAGTAGCGGGGGATAACTACTCGAAAGAGTGGCTA
ATACCGCATACGCCCTACGGGGGAAAGGGGGGGATCGCAAGACCTCTCACTATTGGAGCGG
CCGATATCGGATTAGCTAGTTGGTGGGGTAAAGGCTCACCAAGGCAACGATCCGTAGCTGGT
TTGAGAGGACGACCAGCCACACTGGGACTGAGACACGGCCCAGACTCCTACGGGAGGCAGC
AGTGGGGAATTTTGGACAATGGGGGAAACCCTGATCCAGCCATCCCGCGTGTATGATGAAGG
CCTTCGGGTTCTAAAGTACTTTTGGCAGAGAAGAAAAGGTATCCCCTAATACGGGATACTGCT
GACGGTATCTGCAGAATAAGCACCGGCTAACTACGTGCCAGCAGCCGCGGTAATACGTATGG
TGCAAGCGTTAATCGGAATTACTGGGCGTAAAGCGTGTGTAGGCGGTTCGGAAAGAAAGATG
TGAAATCCCAGGGCTCAACCTTGGAACTGCATTTTTAACTGCCGAGCTAGAGTATGTCAGAG
GGGGGTAGAATTCCACGTGTAGCAGTGAAATGCGTAGATATGTGGAGGAATACCGATGGCG
AATGCAGCCCCCTGGGATAATACTG

Text S3. 16S rRNA sequence of *Acinetobacter calcoaceticus* strain UMTKB-8
GGCTACTGCAGTCGAGCGGAGAGAGGTAGCTTGCTACTGATCTTACGGCGGACGGGTGAGTA
ATGCTTAGGAATCTGCCTATTAGTGGGGGACAACATTTCGAAAGGAATGCTAATACCGCATA
CGTCCTACGGGAGAAAGCAGGGGATCTTCGGACCTTGCGCTAATAGATGAGCCTAAGTCGGA
TTAGCTAGTTGGTGGGGTAAAGGCCTACCAAGGCGACGATCTGTAGCGGGTCTGAGAGGATG
ATCCGCCACACTGGGACTGAGACACGGCCCAGACTCCTACGGGAGGCAGCAGTGGGGAATA
TTGGACAATGGGCGCAAGCCTGATCCAGCCATGCCGCGTGTGTGAAGAAGGCCTTATGGTTG
TAAAGCACTTTAAGCGAGGAGGAGGCTACTTTAGTTAATACCTAGAGATAGTGGACGTTACT
CGCAGAATAAGCACCGGCTAACTCTGTGCCAGCAGCCGCGGTAATACAGAGGGTGCAAGCG
TTAATCGGATTTACTGGGCGTAAAGCGCGCGTAGGCGGCTAATTAAGTCAAATGTGAAATCC
CCGAGCTTAACTTGGGAATTGCATTCGATACTGGTTAGCTAGAGTGTGGGAGAGGATGGTAG
AATTCCAGGTGTAGCGGTGAAATGCGTAGAGATCTGGAGGAATACCGATGGCGAAGGCAGC
CATCTGGCCTAACACTGACGCTGAGGTGCGAAAGCATGGGGAGCAAACAGGATTAGATACC
CTGGTAGTCCATGCCGTAAACGATGTCTACTAGCCGTTGGGGCCTTTGAGGCTTTAGTGGCGC
AGCTAACGCGATAAGTAGACCGCCTGGGGAGTACGGTCGCAAGACTAAAACTCAAATGAAT
TGACGGGGGCCCGCACAAGCGGTGGAGCATGTGGTTTAATTCGATGCAACGCGAAGAACCTT
ACCTGGCCTTGACATAGTAAGAACTTTCCAGAGATGGATTGGTGCCTTCGGGAACTTACATAC
AGGTGCTGCATGGCTGTCGTCAGCTCGTGTCCTGAGATGTTGGGTTAAGTCCCGCAACGAGCG
CAACCCTTTTCCTTATTTGCCAGCGAGTAATGTCGGGAACTTTAAGGATACTGCCAGTGACAA
ACTGGAGGAAGGCGGGGACGACGTCAAGTCATCATGGCCCTTGGCAGGGCTACCACGTGCT
ACAATGGTCGGTACAAGGGTTGCTACCTACCGATGGAAGGCTACCAAAAGCCCATCGATCCG
GATGGGAGTCGGCCCCCCCCCCTGAATTCGAATCCCTTCCGGAACAAAATGCCCGGTGAAAT
TTCCCGGCCTTGAACACCCCCCCGCCCCCCTGGGAGTTTGTTGCCCAAAGATTCCCAATGCAA
AGGGGTACC

Table S1. Retention times and peak areas of the detected monomers for *Sphingobacterium mizutaii* UMTKB-6, *Alcaligenes faecalis* UMTKB-7, and *Acinetobacter calcoaceticus* UMTKB-8.

Strain	Retention time (min)	Peak area	Monomer
Sphingobacterium mizutaii UMTKB-6	5.795	538825.6	CME
	6.358	9937.9	3HHx
	6.771	12703.4	3HO
	7.895	18904.7	3HD
	8.705	27077.2	3HUD
Alcaligenes faecalis UMTKB-7	5.849	652389.1	CME
	7.965	14116	3HD
	4.668	Tr	3HV
	8.774	34397	3HUD
Acinetobacter calcoaceticus UMTKB-8	5.845	444445.1	CME
	7.864	Tr	3HD
	4.600	Tr	3HV
	8.779	12971.3	3HUD

Table S2. Cell dry weight of *Sphingobacterium mizutaii* UMTKB-6, *Alcaligenes faecalis* UMTKB-7, and *Acinetobacter calcoaceticus* UMTKB-8.

Strain	Carbon source	C:N ratio	CDW (g/L)			Average
Sphingobacterium	Sucrose	20	0.0924	0.0850	0.0917	0.0897
mizutaii		30	0.0600	0.9750	0.1015	0.3788
UMTKB-6		40	0.1056	0.0694	0.0936	0.0895
		50	0.0526	0.1117	0.0936	0.0860
Alcaligenes faecalis	Glucose	20	0.0490	0.0230	0.0447	0.0389
UMTKB-7		30	0.0330	0.0587	0.0410	0.0442
		40	0.0340	0.0510	0.0428	0.0426
		50	0.0310	0.0321	0.0395	0.0342
Acinetobacter	Glycerol	20	0.0342	0.0376	0.0628	0.0449
calcoaceticus		30	0.0353	0.0572	0.0504	0.0476
UMTKB-8		40	0.0266	0.0094	0.0098	0.0153
		50	0.0025	0.0019	0.0042	0.0029

Table S3. Primers 341F and 806R targeting 16S rRNA genes of regions V3 and V4 [26].

Host sponge	Barcode sequence	Primer sequence (5′-3′)
X. muta<br>(Bidong Island)	TAGCTT,<br>CGTACG	*F: CCTAYGGGRBGCASCAG<br>R: GGACTACNNGGGTATCTAAT
A. aaptos<br>(Redang Island)	GGCTAC,<br>CGTACG	F: CCTAYGGGRBGCASCAG<br>R: GGACTACNNGGGTATCTAAT
A. aaptos<br>(Bidong Island)	GATCAG,<br>CGTACG	F: CCTAYGGGRBGCASCAG<br>R: GGACTACNNGGGTATCTAAT

* F, forward primer; R, reverse primer.

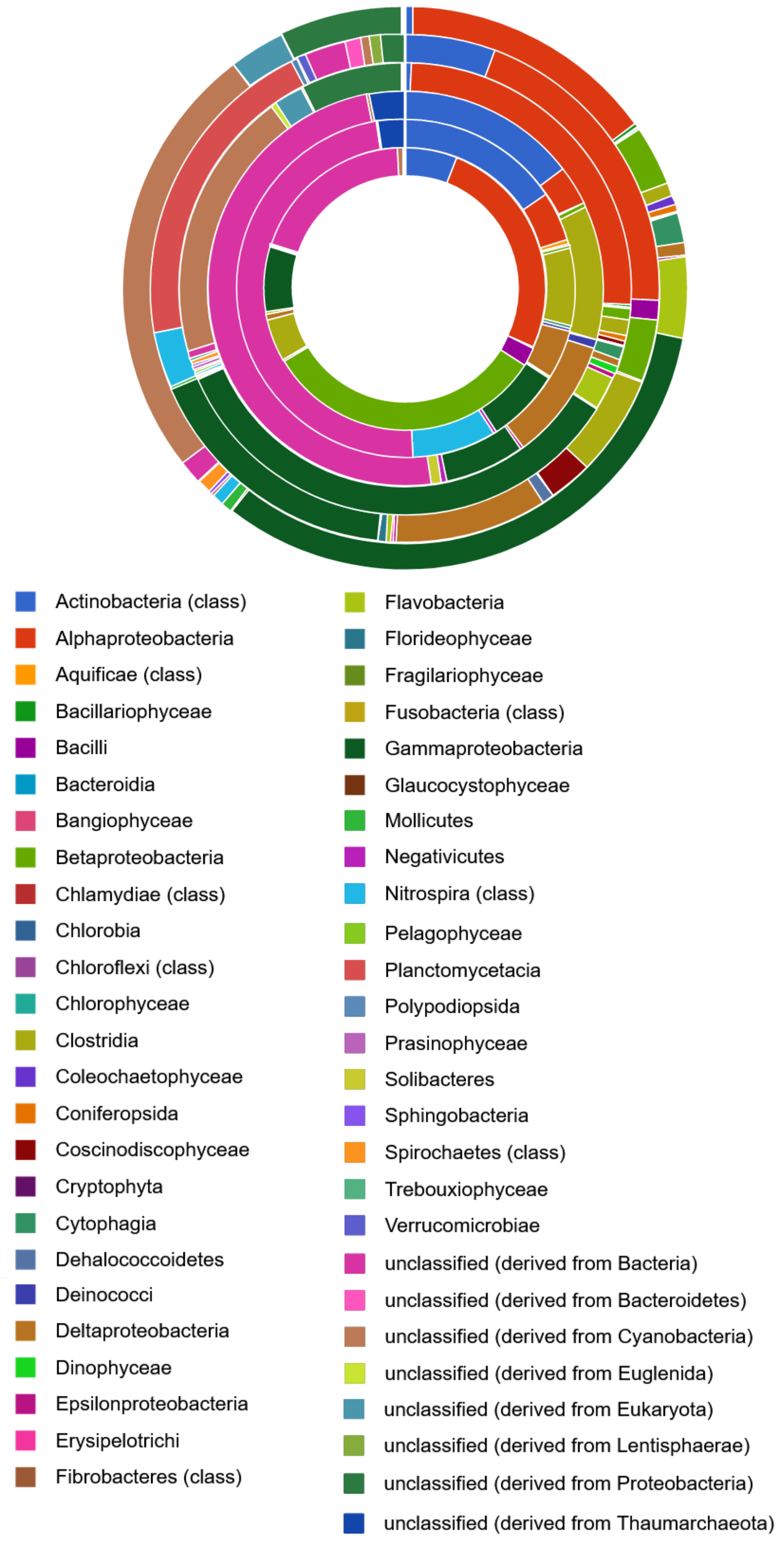

Figure S4. Donut chart showing the relative abundance of classes detected in the bacterial diversity profiles of this study and another similar study accessible via the MG-RAST public repository for comparison [42,43]. Starting from the inner circle are the bacterial communities from 1) *A. aaptos* of Redang Island; 2) *X. muta* of Bidong Island; 3) *A. aaptos* of Bidong Island; 4) *A. corrugata* of Broward County; 5) *A. corrugata* of Fort Lauderdale; 6) *A. corrugata* of Summerland Key.
